# A Comparative Study of Ion Diffusion from Calcium Hydroxide with Various Herbal Pastes through Dentin

**DOI:** 10.5005/jp-journals-10005-1405

**Published:** 2017-02-27

**Authors:** Priyanka Dausage, Rajesh B Dhirawani, Jayant Marya, Vrinda Dhirawani, Vijayendra Kumar

**Affiliations:** 1 Consultant, Department of Pedodontics and Preventive Dentistry, Jabalpur Hospital & Research Centre, Jabalpur, Madhya Pradesh, India; 2 Professor and Head, Department of Oral and Maxillofacial Surgery, Hitkarini Dental College & Hospital, Jabalpur, Madhya Pradesh, India; 3 Postgradute Student, Department of Oral and Maxillofacial Surgery, Hitkarini Dental College & Hospital, Jabalpur, Madhya Pradesh, India; 4 Postgradute Student, Department of Conservative Dentistry, Hitkarini Dental College & Hospital, Jabalpur, Madhya Pradesh, India; 5 Reader, Department of Oral and Maxillofacial Surgery, Malla Reddy Institute of Dental Sciences, Hyderabad, Telangana, India

**Keywords:** Calcium hydroxide, Herbal paste, Intracanal medicament, Ion diffusion.

## Abstract

**Aim:**

The aim of this study was to evaluate the diffusion ability of ions through dentinal tubules of different nonalcoholic calcium hydroxide-containing herbal pastes and compare it with the calcium hydroxide paste prepared with saline.

**Materials and methods:**

A total of 36 single-rooted premolar teeth were used in this study. The tooth crowns were removed and the root canals were prepared. Depending on the vehicle to be used for preparing calcium hydroxide pastes, six groups were made: Group I: Ca(OH)_2_ saline paste (control group), group II: Ca(OH)_2 _papaya latex paste, group III: Ca(OH)_2_ coconut water paste, group IV: Ca(OH)_2_ Ashwagandha *(Withania somnifera)* paste, group V: Ca(OH)_2_ Tulsi *(Ocimum tenuiflorum)* paste, and group VI: Ca(OH)_2 _garlic *(Allium sativum)* paste. After biomechanical preparation, calcium hydroxide herbal paste dressings were applied and sealed with resin-based cement. The teeth were placed in containers with deionized water, and the pH of the water was measured at regular intervals over 3, 24, 72, and 168 hours.

**Results:**

We observed that all herbal pastes allowed the diffusion of ions, but pastes prepared with Ashwagandha and papaya latex showed more ion diffusion after 168 hours and marked increase in pH, depicting better support for calcium hydroxide action.

**Conclusion:**

We conclude that Ashwagandha and papaya latex allow better diffusion of calcium hydroxide through den-tinal tubules, thus enhancing its action, and advise its use as a vehicle for placing intracanal medicament.

**How to cite this article:**

Dausage P, Dhirawani RB, Marya J, Dhirawani V, Kumar V. A Comparative Study of Ion Diffusion from Calcium Hydroxide with Various Herbal Pastes through Dentin. Int J Clin Pediatr Dent 2017;10(1):41-44.

## INTRODUCTION

Antimicrobial intracanal medicaments are used to complement the disinfection of the root canal system. Calcium hydroxide [Ca(OH)_2_] has been widely used for its biological and antimicrobial ability, to dissolve organic tissues, and the capacity to inactivate bacterial endotox-ins. The effectiveness of Ca(OH)_2_-based pastes depends on diffusion of hydroxyl ions [OH^-^] in concentrations to reach adequate pH levels.^1,2^

The success of calcium hydroxide paste as a root canal dressing is related to its dissociation into calcium and hydroxyl ions.^3^ Hydroxyl ions [OH^-^] are highly reactive free radicals that show extreme reactivity with several biomolecules. This reactivity is indiscriminate, so this free radical rarely diffuses away from the sites of generation. The lethal effects of hydroxyl ions in bacterial cells are due to the damage of bacterial cytoplasmic membrane, protein denaturation, and damage to the bacterial deoxyribonucleic acid.^4^

The Ca(OH)_2_ powder for root canal dressing has been used with different vehicles, such as distilled water, camphorated monochlorophenol, normal saline, cresatin, glycerine, and propylene glycol (PG). The dissociation of Ca(OH)_2_ into OH^-^ and Ca^2^+ depends on the vehicle used to prepare the paste, and it should allow gradual and efficient dissociation of hydroxyl ions for better action of calcium hydroxide.^5,6^ Studies have demonstrated that the vehicle can exert a great influence on the release of ions.^7^

Thus, several associations have been investigated to determine the effectiveness of calcium hydroxide pastes. Besides allowing dissociation of the calcium hydroxide, the vehicle also should enhance the antimicrobial capacity of the paste. According to Gomes et al,^7^ chlorhexidine may be used as a vehicle in an attempt to increase the antimicrobial capacity of calcium hydroxide pastes, as it is effective against Gram-positive and Gram-negative, aerobic and facultative anaerobic microorganisms, yeasts, and viruses. Based on these aspects, this study evaluated the ability of calcium hydroxide mixed with different vehicles to diffuse through dentin.^8^

Considering the antimicrobial potential of a herbal-calcium hydroxide association, it becomes important to determine how well such pastes can promote the diffusion of ions through dentinal tubules, an attribute essential to the therapeutic effect of any calcium hydroxide paste.

## MATERIALS AND METHODS

The present study was carried out in the Department of Pedodontics and Preventive Dentistry, Hitkarini Dental College and Hospital, Jabalpur, Madhya Pradesh, India, and was approved by the institutional ethical committee.

The study was done on 36 extracted single-rooted premolar teeth with the following criteria:

### Inclusion Criteria

- Single-rooted premolar teeth
- Teeth with no gross caries or fracture
- Teeth having patent canals
- Teeth with full length of root intact.

### Exclusion Criteria

- Teeth with gross deviation in their normal anatomy
- Teeth with internal resorption and external resorption
- Teeth having fractured roots
- Teeth with obstruction or calcification within the canal system were excluded from this study.

### Specimen Preparation^3^

A total of 36 extracted human single-rooted premolar teeth were collected, stored, and disinfected (10% formaldehyde) and handled as per the recommendation and guidelines laid down by the Centers for Disease Control. The crowns were transversely sectioned with a carborundum disk (Ukam, USA), at the cementoenamel junction level. Root canal length was measured by inserting a #15 K-file (Dentsply, Petropolis, Brazil) with a rubber stop. When the file tip reached the apical foramen, the stop was leveled to the cervical edge of the root, and the canal length was recorded. The working length was established by subtracting 1 mm from the total root canal length. Apical preparation was performed up to this limit, up to file #40. The root canals were irrigated with distilled water throughout the instrumentation procedure with a 2-mL disposable syringe. After instrumentation, a #40 file was inserted to the total working length for apical cleaning, and the root canal was filled with an ethylenediamine-tetraacetic acid solution (Healix Medico, Mumbai) for 3 minutes. After this period, the root canals were rinsed with saline solution and dried with absorbent paper points (Dentsply, Petropolis, Brazil).

### Sample Size and Distribution

The teeth were randomly divided into six groups according to the following calcium hydroxide pastes used as an intracanal medicament:

- *Group I (n = 6):* Calcium hydroxide-saline paste, prepared by mixing 1 gm of calcium hydroxide with 2 mL of saline.
- *Group II (n = 6):* Calcium hydroxide-papaya latex paste, prepared by mixing 1 gm calcium hydroxide with 2 mL of papaya latex extract.
- *Group III (n = 6):* Calcium hydroxide-coconut water paste, prepared by mixing 1 gm calcium hydroxide with 2 mL of coconut water.
- *Group IV (n = 6):* Calcium hydroxide-Ashwagandha paste, prepared by mixing 1 gm calcium hydroxide with 2 mL of Ashwagandha extract.
- *Group V (n = 6):* Calcium hydroxide-Tulsi paste, prepared by mixing 1 gm calcium hydroxide with 2 mL of Tulsi extract.
- *Group VI (n = 6):* Calcium hydroxide-garlic paste, prepared by mixing 1 gm calcium hydroxide with 2 mL of garlic extract.

### Preparation of Extracts

Papaya Latex Extract^9^

About 10 gm of dried papaya latex was crushed with 100 mM phosphate buffer (pH = 5.5) using acid-washed sand in a pestle mortar in chilly conditions for 10 to 15 min. The mixture was centrifuged at 10,000 rpm for 10 min. The pellet was discarded and the supernatant was kept. To the supernatant, solid ammonium sulfate was added to achieve 45% saturation at 4°C. After cen-trifugation, the pellet was kept and was redissolved in phosphate-buffered saline (1 mL).

Tulsi Leaves, Ashwagandha, Garlic Extract^10^

About 10 gm of material (dried Tulsi leaves, dried Ash-wagandha powder, and chopped garlic cloves) was taken into the thimble of Soxhlet apparatus. The extraction was started using 250 mL of ethanol. The extraction was continued up to 10 cycles (10 gm of material extracted with 250 × 10 = 2.5 L ethanol). The extract obtained was dried under vacuum and redissolved in 1 mL saline before use.

Coconut Water

Coconut water was used as obtained from green coconut and was taken fresh.

Complete filling of the root canals was checked by having the solution overflow through the apical foramen and flow back through the root canal opening. After the complete filling of the root canals, their openings were sealed with temporary cement (Cavit, 3M ESPE™). The apical foramen and root canal apex (over the temporary cement) were sealed with epoxy resin (RelyX, 3M ESPE™, India). Next, the teeth were placed in containers with 50 mL of deionized water (pH = 6.17) and kept in an oven at 37°C, with 100% humidity.

After 3, 24, 72, and 168 hours, the pH values of the solutions in the containers were measured using a pH meter (Digital - 1010 *Esico,* India), calibrated and standardized with standard buffer solutions (Merck, Germany) at pH 4.00 and 7.00. Twelve measurements were performed for each group/period. For each measurement, the electrode of the pH meter was carefully rinsed with deionized water and dried with absorbent paper to eliminate any residues that could interfere with the measurements. The data were recorded in tables, and the differences between groups and periods were statistically analyzed by one-way analysis of variance (ANOVA) and Tukey’s *post hoc* comparisons using Sigma GraphPad Prism^®^ version 6.0.

## RESULTS

In the present study, 36 single-rooted premolar teeth were used and divided into six groups, based on different vehicles used to place calcium hydroxide. At regular intervals of 3, 24, 72, and 168 hours, the pH of the water was recorded for each group.

**Graph 1:**
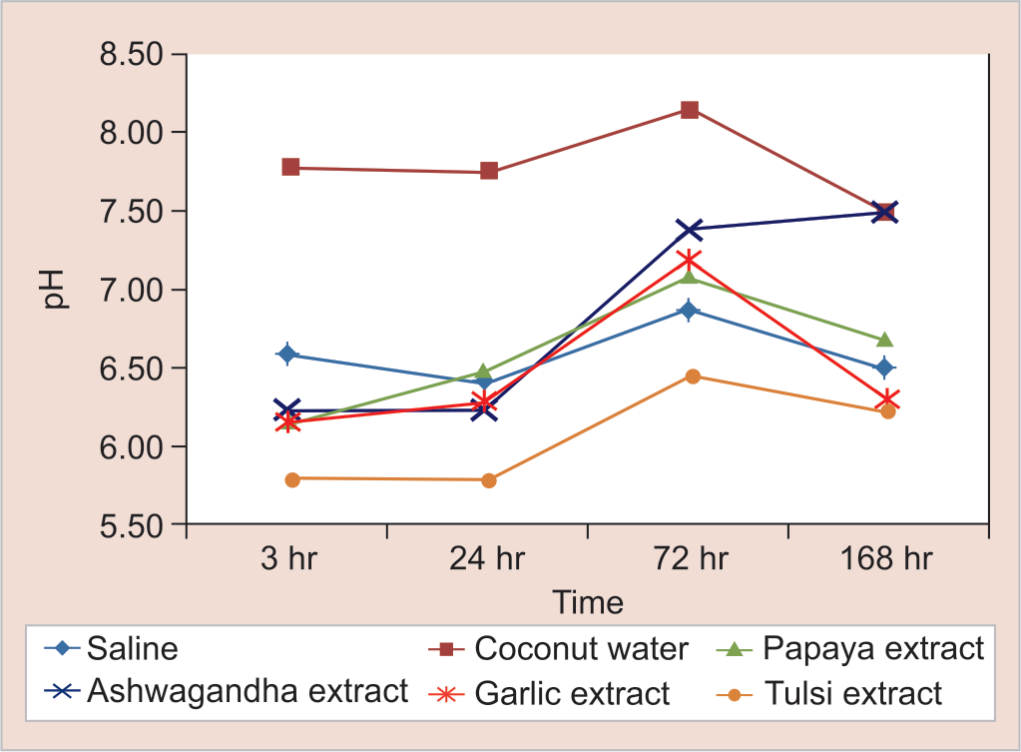
pH variation over time for the study groups

Graph 1 suggests that all the herbal pastes caused the increase in pH up to 72 hours, and then there is a marked decrease in the pH with all the herbal preparations. Coconut water was alkaline in nature initially and after 168 hours, the medium was still alkaline. Ashwagandha was the only herbal preparation that increased the pH up to 168 hours and made the medium alkaline. All the other preparations were acidic at the start of experiment, increased the pH up to 72 hours, but could not make the medium alkaline. A reduction in pH after 168 hours was seen with all the extracts.

Table 1 shows the mean pH values of all groups. Group I was used as a control group, in which saline was mixed with calcium hydroxide, and showed a pH drift ranging from 6.6 to 6.8 with the mean pH of 6.6 for 3 hours. No statistically significant difference was observed for the pH change during 24, 72, and 168 hours pH measurement (p < 0.05).

In group II, papaya latex was used and showed initial mean pH as 6.14. The pH was increased with time and was statistically different from that of pH at 3 hours after 72 hours and 168 hours.

Group III used coconut water mixed with calcium hydroxide and recorded mean pH of 7.78, which increased significantly to 8.15 after 72 hours and decreased during 168 hours measurement.

Group IV used Ashwagandha paste and its mean pH value was increased significantly to 8.08 after 72 hours, as compared with 6.14 after 3 hours. The 168 hours pH value, though decreased to 7.67, was still significantly different from the 3 hours pH value.

Group V was Tulsi paste mixed with calcium hydroxide and showed a significant increase of the mean pH to 6.45 after 72 hours from initial pH as 5.8.

Group VI used garlic paste and showed a significant increase in pH as 7.2 from initial pH as 6.15 after 72 hours.

Although all the herbal preparations showed an increase in the pH of the solution upto 72 hours, only papaya and Ashwagandha paste did not allow the decrease of the pH after 168 hours, and the mean pH values were still significantly different from that of the mean pH at 3 hours thus, inferring that they allow the maximum ionization of calcium hydroxide and promote sustained release of the ions for a longer duration of time.

**Table 1:** **Table 1:** Comparison of mean pH values between groups at different time intervals

*Groups*		*Materials*		*3 hours*		*24 hours*		*72 hours*		*168 hours*	
I		Ca(OH)_2_ + saline		6.60 ± 0.44^a^		6.30 ± 0.55^a^		6.81 ± 0.47^a^		6.51 ± 0.37^a^	
II		Ca(OH)_2_ + papaya		6.14 ± 0.30^a^		6.50 ± 0.53^a^		7.08 ± 0.58^b^		6.67 ± 0.4^b^	
III		Ca(OH)_2_ + coconut		7.78 ± 0.56^a^		7.74 ± 0.41^a^		8.15 ± 0.11^b^		7.47 ± 0.31^a^	
IV		Ca(OH)_2_ + ashwagandha		6.14 ± 0.30^a^		6.50 ± 0.53^a^		8.08 ± 0.58^b^		7.67 ± 0.4^b^	
V		Ca(OH)_2_ + tulsi		5.80 ± 0.37^a^		5.81 ± 0.25^a^		6.45 ± 0.06^b^		6.21 ± 0.43^a^	
VI		Ca(OH)_2_ + garlic		6.15 ± 0.11^a^		6.28 ± 0.15^a^		7.20 ± 0.17^b^		6.28 ± 0.12^a^	

Different letters in superscript shows the statistically different values after one-way ANOVA (p < 0.05)

## DISCUSSION

Calcium hydroxide is one of the main root canal dressings used in endodontics. The success of calcium hydroxide as a root canal dressing lies in its dissociation into ionic forms, i.e., calcium and hydroxyl ions. These hydroxyl ions alkalinize the environment and thus help in activating the alkaline phosphatase enzyme, which induces mineralized tissue formation and thus is helpful in the repair process.^4^

To be effective, the hydroxyl ions should be able to diffuse in dentin and remain in pulp tissues at a sufficient concentration to produce the pH level required to destruct bacteria inside the root canal and dentinal tubules. The action of these ions on tissues and bacteria explains the biological and antimicrobial properties of calcium hydroxide.^11^

Calcium hydroxide is used as paste, and the vehicle required to make the paste of calcium hydroxide may well increase the suitability of calcium hydroxide as a filling.^12^ The studies have proved that aqueous and viscous vehicles have more effectiveness as compared with the oily vehicles.^2^ Similarly, it has been shown that nonalcoholic preparations of PG and bee propolis as vehicles showed good diffusion through dentin.^3^

The present study was aimed to identify some more preparations to be used as a vehicle for calcium hydroxide paste to improve the diffusion capabilities of calcium hydroxide. Aqueous vehicles were used in the present study, which was in accordance with the studies conducted previously.^11^ Herbal preparations are always welcome in present-day science due to the multitude of benefits due to the phytochemicals present in herbs. Withaferin A and 3-b-hydroxy-2,3-dihydro-withanolide F isolated from Ashwagandha *(Withania somnifera)* show promising antibacterial, antitumor, immunomodulating, and anti-inflammatory properties.^13^ Such herbal preparations, if used smartly, can provide additional benefits. In the present study, Ashwagandha and the papaya latex showed the diffusion of ions through the dentinal tubules in a sustained manner (up to 168 hours) in comparison with normal saline. All the herbal pastes in the present study, which diffused through the dentinal tubules, presented a better ability to alkalize external root surface after 72 hours when compared with saline.

Coconut water has a much better composition of minerals like calcium, iron, manganese, magnesium, and zinc compared with other fruits.^14^ The better diffusion ability and marked antimicrobial activity make them the best materials, which can be used as vehicles. The use of papaya latex extract and Ashwagandha with calcium hydroxide as an intracanal medicament may thus be suggested.

## CONCLUSION

Ashwagandha and papaya latex extracts as vehicles for calcium hydroxide are suggested as an intracanal medicament. Further studies are required to confirm the present data.
